# Impaired Folate-Mediated One-Carbon Metabolism in Type 2 Diabetes, Late-Onset Alzheimer’s Disease and Long COVID

**DOI:** 10.3390/medicina58010016

**Published:** 2021-12-23

**Authors:** Melvin R. Hayden, Suresh C. Tyagi

**Affiliations:** 1 Departments of Internal Medicine, Endocrinology Diabetes and Metabolism Diabetes and Cardiovascular Disease Center, University of Missouri School of Medicine, Columbia, MO 65212, USA; 2 Department of Physiology, University of Louisville School of Medicine, Louisville, KY 40202, USA; suresh.tyagi@louisville.edu

**Keywords:** Alzheimer’s disease, COVID-19, epigenetics, homocysteine, Long COVID, MTHFR gene mutations, nutritional B vitamin deficiencies, PASC, repurposed drugs, type 2 diabetes mellitus

## Abstract

Impaired folate-mediated one-carbon metabolism (FOCM) is associated with many pathologies and developmental abnormalities. FOCM is a metabolic network of interdependent biosynthetic pathways that is known to be compartmentalized in the cytoplasm, mitochondria and nucleus. Currently, the biochemical mechanisms and causal metabolic pathways responsible for the initiation and/or progression of folate-associated pathologies have yet to be fully established. This review specifically examines the role of impaired FOCM in type 2 diabetes mellitus, Alzheimer’s disease and the emerging Long COVID/post-acute sequelae of SARS-CoV-2 (PASC). Importantly, elevated homocysteine may be considered a biomarker for impaired FOCM, which is known to result in increased oxidative–redox stress. Therefore, the incorporation of hyperhomocysteinemia will be discussed in relation to impaired FOCM in each of the previously listed clinical diseases. This review is intended to fill gaps in knowledge associated with these clinical diseases and impaired FOCM. Additionally, some of the therapeutics will be discussed at this early time point in studying impaired FOCM in each of the above clinical disease states. It is hoped that this review will allow the reader to better understand the role of FOCM in the development and treatment of clinical disease states that may be associated with impaired FOCM and how to restore a more normal functional role for FOCM through improved nutrition and/or restoring the essential water-soluble B vitamins through oral supplementation

## 1. Introduction

Impaired folate-mediated one-carbon metabolism (FOCM) is an emerging topic in type 2 diabetes mellitus (T2DM), late-onset Alzheimer’s disease (LOAD) and the emerging post-viral syndrome of long coronavirus disease-19 (Long COVID) (LC)—post-acute sequelae of severe acute respiratory syndrome coronavirus 2 (SARS-CoV-2) (PASC). Hereafter, the term Long COVID-PASC will be referred to as LC/PASC. This review will focus on the above two clinical diseases and the post-viral syndrome of LC/PASC and discusses the role of impaired FOCM in three sections as follows: Section 2, impaired FOCM in T2DM; Section 3, impaired FOCM in LOAD; and Section 4, impaired FOCM in LC/PASC. Each of these sections will also discuss the role of homocysteine (Hcy) and hyperhomocysteinemia (HHcy) since HHcy is currently known to be associated with increased oxidative–redox stress (increased reactive oxygen–nitrogen species). Importantly, HHcy is an independent risk factor for cerebro-cardiovascular disease (CVD) [1] as well as a biomarker of impaired FOCM [2,3,4].

There has been renewed interest in FOCM during the past several years due to recent insights, which indicate dietary inadequacies of micronutrients or genetic polymorphisms. These dietary micronutrient deficiencies include the essential water-soluble vitamins B9 (folate-folic acid), vitamin B12 and vitamin B6. Importantly, these vitamin deficiencies are closely linked to CVD, vascular abnormalities including endothelial activation and dysfunction, myocardial diseases (myocardial infarction and abnormal remodeling in congestive heart failure), hypertension, stroke, LOAD, T2DM, neural tube defects, epigenetic abnormalities and cancer [5].

Hcy is a nonessential (non-proteinogenic) sulfur-containing amino acid and an intermediary metabolic product derived from the demethylated essential amino acid methionine [6]. It is commonly known that plasma concentrations of Hcy are inversely related to plasma concentrations of folate, vitamin B12, and vitamin B6, as well as to the intake of these vitamins [6,7,8,9,10,11]. Further, it is now accepted that HHcy has vasculotoxic effects as well as neurotoxic effects that are associated with neuroinflammation, neurodegeneration, pro-oxidation as well as proatherogenic/prothrombotic effects [6,7,8,9,10,11,12]. HHcy plays an important role in the causation of oxidative stress with excess formation of reactive oxygen nitrogen species (RONS) in the vascular endothelium and multiple organ systems due to autoxidation of Hcy, formation of Hcy mixed disulfides, interactions of Hcy thiolactones and protein homocysteinylation [6,12,13,14,15].

**Figure 1:**
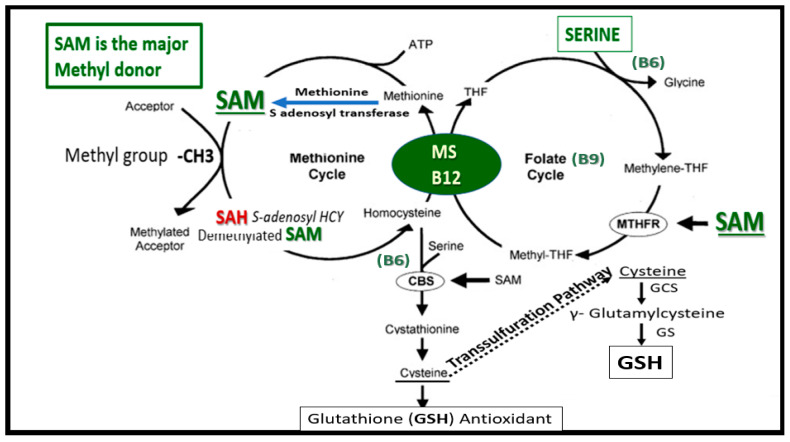
Folate-Mediated One-Carbon Metabolism (FOCM). This figure illustrates both the folate and methionine interdependent cycles and supports the importance of the methyl donor S-adenosylmethionine (SAM) as well as demonstrating the importance of the essential B vitamins. Importantly, note that methionine and tetrahydrofolate (THF) are derived primarily through dietary intake to supply the methionine and folate cycles and that the enzyme methionine synthase (MS) and its essential cofactor vitamin B12 are placed in a central position of the interconnected folate and methionine cycles. FOCM comprises a network of interconnected folate-dependent metabolic pathways responsible for serine and glycine interconversion, de novo purine synthesis, de novo thymidylate synthesis and homocysteine remethylation to methionine as well as providing antioxidant defense via glutathione (GSH) production via the transsulfuration pathway. Note that the encircled methylenetetrahydrofolate reductase (MTHFR) enzyme plays and important role in the folate cycle. The most common genetic variant in MTHFR gene to date is the 677C > T polymorphism, which results in elevated levels of Hcy especially if there is deficient folate. Once Hcy is synthesized through multiple steps in the methionine cycle, it may then undergo remethylation to methionine or be eliminated through the transsulfuration pathway. Additionally, thymidylate synthase (TYMS) converts deoxyuridine monophosphate (dUMP) to deoxythymidine monophosphate (dTMP) (not shown) in a 5,10-methylene-THF-dependent reaction. Importantly, cystathionine beta synthase (CBS) and cystathionine gamma lyase (CSE-CGL) do not only contribute to generate GSH (antioxidant) in the transsulfuration pathway but also are important for endothelial cell generation of hydrogen sulfide (H_2_S), a known gaso-transmitter and vasodilator. Elevation of Hcy from the methionine cycle may result in hyperhomocysteinemia, which is an independent risk factor for cerebro*-*cardiovascular diseases, accelerated atherosclerosis, thromboembolism, hypoxemia and stroke. *CBS = cystathionine-beta-synthase; GCS = glutamate cysteine ligase (gamma-glutamylcysteine synthetase); GS = glutathione synthase; GSH = glutathione; MTHFR = methylenetetrahydrofolate; MS = methionine synthase; THF = tetrahydrofolate*.

**Figure 2:**
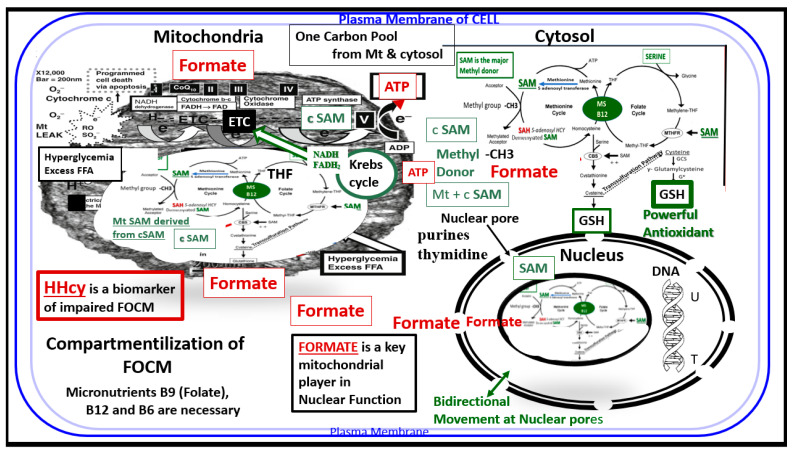
Compartmentalization of FOCM. Note the presence of the folate-methionine one carbon cycle metabolism in the cytoplasm (cytosol), mitochondria and nucleus. Additionally, note the importance of formate being transferred from the mitochondria to the nucleus, as well as *S*-adenosylmethionine (SAM) via nuclear pores. Importantly, deoxythymidine monophosphate (dTMP) synthesis occurs in the cytosol, nucleus and mitochondria, whereas purine synthesis and methionine synthesis take place within the cytosol. Mitochondrial FOCM generates formate for cytosolic and nuclear FOCM and biosynthetic precursors for mtDNA synthesis and mitochondrial protein translation. Thymidylate synthase (TYMS) converts deoxyuridine monophosphate (dUMP) to dTMP in a 5,10-methylene-THF-dependent reaction (not shown). It is important to note that mitochondrial SAM (Mt SAM) is derived from cytosolic SAM (cSAM). Additionally, the Krebs cycle also resides within the mitochondria and provides NADH and FADH2 to the electron transport chain for ATP production. *ATP = adenosine triphosphate; c = cytosol; ETC = electron transport chain; FAD = flavin adenine dinucleotide; FADH = reduced flavin adenine dinucleotide; FFA = free fatty acids; HHcy = hyperhomocysteinemia; MS = methionine synthase; Mt = mitochondria; NADH = reduced nicotinamide adenine dinucleotide; T = thymidylate-thymine; U = uracil*.

FOCM is known to support multiple physiological processes. These include biosynthesis of purines and thymidylate, amino acid homeostasis of glycine, serine and methionine, epigenetic maintenance as well as providing antioxidant defense via glutathione (GSH) against RONS. Additionally, FOCM is also important in the generation of energy via adenosine triphosphate (ATP) generation in the mitochondria (Figure 1 and Figure 2).

Additionally, the folate and methionine cycles are compartmentalized in cells and exist in the cytoplasm (cytosol), mitochondria and nucleus [16] (Figure 2)

Folate is necessary for FOCM, which involves an essential network of pathways involved in the transfer and eventual utilization of one-carbon units. This network is necessarily required for both DNA and RNA biosynthesis, amino acid metabolism, and methylation reactions. Deficient folate is most often related to the deficiency of the vitamins B9, B12 and B6. Note that when tetrahydrofolate (THF) enters the folate one-carbon cycle it acquires a carbon unit from serine (vitamin B6–dependent) to form 5,10 methyleneTHF, and once this is generated it follows along the folate cycle to result in various fates as in Figure 1 and Figure 2. Folate may also serve for conversion to 5-methyltetrahydrofolate (5-MTHF), or be utilized as the one-carbon donor in the synthesis of nucleic acids, where it is required by thymidylate synthetase in the conversion of deoyxuridine to deoxythymidine for pyrimidine biosynthesis or it may be converted to other metabolites for folate dependent purine biosynthesis [17,18]. While the focus in this review is primarily on B9, B12, and B6 there are also eight known B vitamins consisting of B1 *thiamine*, B2 *riboflavin*, B3 *nicotinamide*, B5 *pantothenic acid*, B6 *pyridoxine*, B7 *biotin*, B9 *folate* and B12 *cobalamin* that are important and necessary for proper homeostasis and health.

## 2. Impaired FOCM in T2DM

T2DM is a multifactorial polygenic disease that may be characterized as a chronic metabolic–endocrine disorder that associates with insulin resistance or relative lack of insulin or insulin deficiency and thus hyperglycemia. The resulting glucotoxic state promotes RONS stress and chronic inflammation [19].

Importantly, T2DM is an independent risk factor for macrovascular (accelerated atherosclerosis and vascular stiffness) and microvascular complications, which includes multiple diabetic-opathies such as retinopathy [20], neuropathy [21], nephropathy [20,21,22], vasculopathy—intimopathy [23], isletopathy [24], atheroscleropathy [25] and diabetic cognopathy (cognitive dysfunction) [26], which is associated with an increased risk for age-related neurodegenerative diseases such late onset Alzheimer’s disease (LOAD) [27].

**Figure 3:**
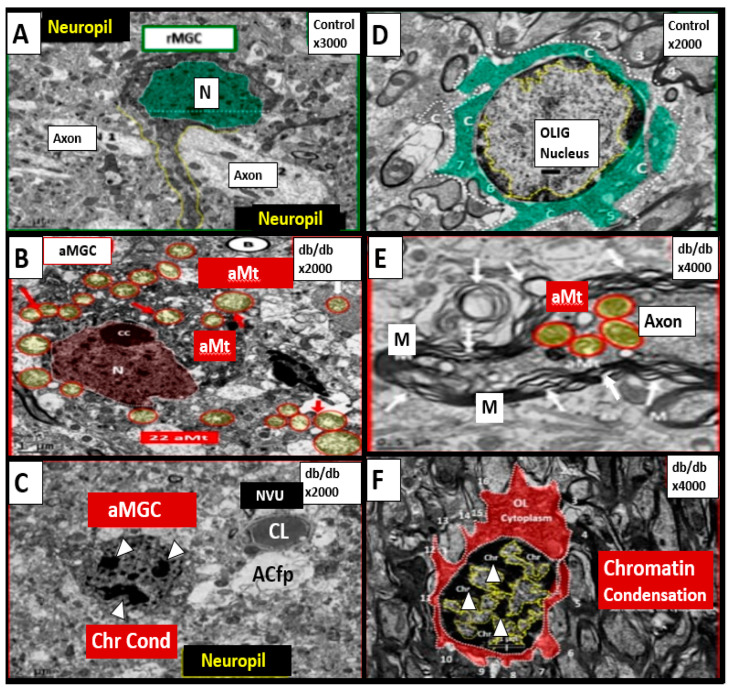
Chromatin Condensation in Aberrant Microglia and Oligodendrocytes in Diabetic Female db/db Models in Grey Matter—Cortical Layer III. This multipanel collage illustrates chromatin condensation in aberrant activated microglia cell(s) (aMGC) and aberrant oligodendrocytes (OL-OLG). Panels (**A**,**D**) illustrate the normal microglia cell (MGC) and oligodendrocyte in control non-diabetic models respectively. Note the abnormal aberrant mitochondria (aMt) in the microglia and neural cells in panels (**B**,**E**) respectively with aMt (highlighted yellow circles encircled in red). Panels (**C**,**F**) depict chromatin condensation (Chr Cond) within the nuclei of aberrant microglial cells and oligodendrocytes respectively. Also, note arrows in panel E depict myelin splitting and separation and arrowheads depict chromatin condensation in the nuclei (panels (**C**,**F**)). aMGCs and aberrant oligodendrocytes suggest abnormal crosstalk with abnormal myelin remodeling and impaired folate one-carbon metabolism. Magnification and scale bar varies. Images in this figure were provided and approved by CC 4.0 [32,33]. *M = myelin; N = nucleus; NVU = neurovascular unit; Ol-Olig = oligodendrocyte*.

T2DM is also associated with elevations of Hcy (HHcy) and thus, is associated with increased oxidative–redox stress as well as impaired FOCM [6,28,29,30,31]. Indeed, in the insulin resistant obese diabetic female db/db models there appears to be a strong and recurrent association of aberrant mitochondria (Mt) with enlargement and a loss of Mt matrix electron density with increased lucency and loss of crista that is associated with chromatin condensation in activated microglia and oligodenrocytes (Figure 3) [27,32,33,34].

**Figure 4:**
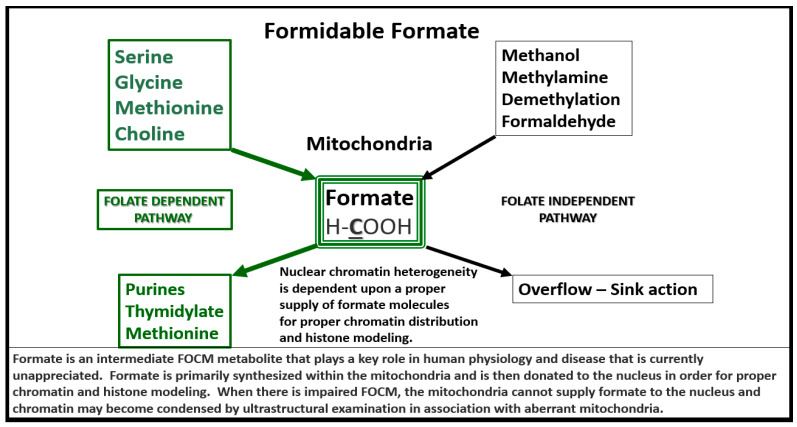
Formate Plays a Central and Formidable Role in Providing Proper Nucleus Function and Structure. Serine, glycine, methionine and choline are also necessary components to fulfill proper mitochondrial function to the nucleus in order to produce purines, thymidylate and methionine to fulfil their role in the nucleus. This figure depicts the importance of the folate dependent pathway (green coloring) for formate synthesis in the mitochondria and the central role for the formidable formate to be utilized by the nucleus for proper chromatin modeling. Note the bold and underlining of the important carbon unit in the chemical formula for formate. Also, note the folate independent pathway on the right-hand side of this figure. The concept for this slide design was derived from reference [35].

Importantly, formate is primarily produced in the mitochondria and then secreted to enter the nucleus via nuclear pores in health to provide FOCM to the nucleus for proper chromatin, histone modeling and normal function (Figure 4) [35].

Therefore, aberrantly remodeled mitochondria as in the diabetic db/db models will not be able to produce formate and chromatin condensation ensues as noted in Figure 3. Importantly, Santos et al. recently demonstrated that deficiency of glycine (one of the precursors to formate in Figure 4) resulted in hydrocephalus—ventriculomegaly in glycine decarboxylase-deficient mice [36]. This is another finding suggesting the important role of FOCM and deficient formate involving abnormal brain structure. This group also suggested that impaired enzymatic activity within mitochondrial FOCM, as opposed to diminished exogenous supply or a methyl trap, can be a direct cause of aqueduct stenosis (the most commonly known cause of congenital hydrocephalus in humans).

The above findings regarding chromatin condensation in a diabetic model suggest an important role for FOCM in health and that impairment of FOCM is detrimental to physiologic homeostasis and normal development. Thus, impaired FOCM may play an important role in T2DM as more research is fostered in this exciting field of study. Additionally, Zhao et al. have recently shown that chronic folate deficiency induces glucose and lipid metabolism disorders and subsequent cognitive dysfunction in mice [37]. These authors also pointed out that previous studies have shown lower folate levels and higher Hcy levels in patients with T2D as compared with non-diabetic subjects [38,39]. Therefore, the involvement of impaired FOCM in T2DM seems to be viewed through the lens of HHcy, increased oxidative—redox stress and abnormal remodeling of mitochondria and nuclear chromatin condensation.

### 2.1. The Neurovascular Unit (NVU) in Diabetic Brain of the db/db Mouse

**Figure 5:**
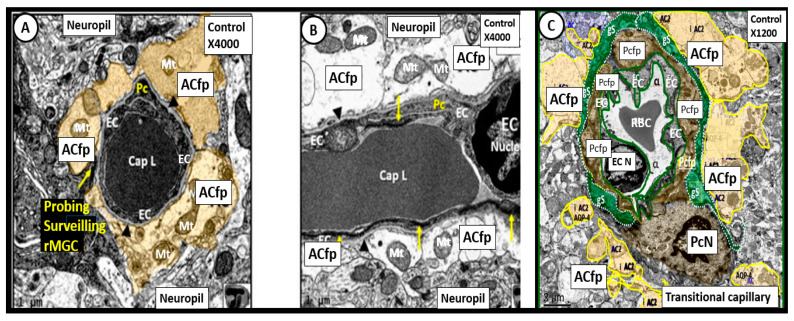
Neurovascular Unit Capillary (NVU) in control non-diabetic models. Panels (**A**) (cross section), (**B**) (longitudinal section) and (**C**) (cross section) depict the normal NVU composed of the endothelial cell (EC), pericyte foot processes (Pcfp), astrocyte foot processes (ACfp) and the ramified microglia cell (rMGC). Note in panel (**A**) that the rMGC is probing the NVU to surveil for any injury to the NVU. Note how tightly the Pcfp and the ACfp adhere and abut to the basement membrane of the ECs. Additionally, note the ACfp are pseudo colored yellow in panels (**A**,**C**) and reveal their electron lucent cytoplasm in panel (**B**), and the nanometer glymphatic space (gS) is pseudo colored green in panel (**C**). Varying magnifications upper right of each panel. Images are reproduced and modified by CC 4.0 [40].

In our electron microscopy core laboratory, we have previously utilized the hyperleptinemic obese, insulin resistant and diabetic female *db/db* mouse models of diabetes in order to study the remodeling effects of diabetes in the brain [27,32,33,34]. These experiments in the *db/db* model have revealed a marked remodeling of the NVU. The cellular content of the control non-diabetic NVU includes the following four cells: the endothelial (EC), pericyte (Pc) and its foot processes (Pcfp), astrocyte (AC) and its foot processes (ACfp) and the microglia cell(s) (MGC) (Figure 5).

**Figure 6:**
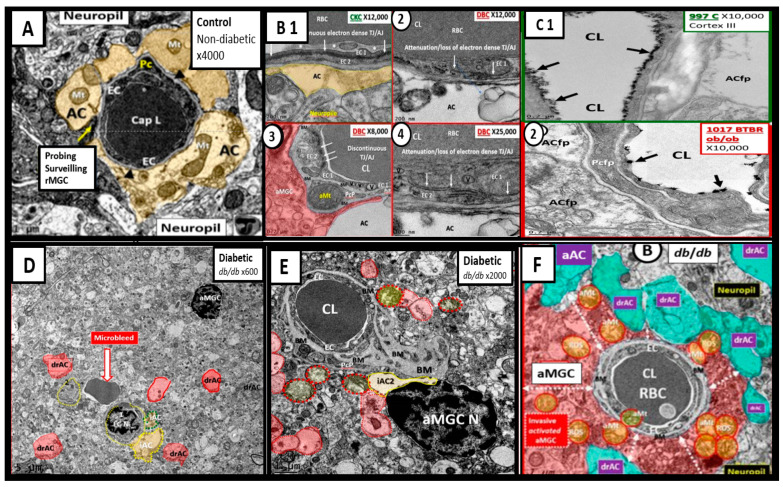
Compilation of Abnormal Remodeling in the Cortical Grey Matter Layer III in Diabetic *db/db* and BTBR *ob/ob* Neurovascular Unit (NVU). Panel (**A**) is the control model and depicts the normal morphology of the NVU and the remainder of images are compiled from the diabetic *db/db* and BTBR *ob/ob* models. Panel (**B1**) (control with intact blood-brain barrier (BBB)) and (**2**–**4**) depicts the attenuation and or loss of the endothelial cell (EC) tight and adherens junctions (TJ/AJ) of the BBB. Panel (**C1**) depicts the highly electron dense endothelial glycocalyx (arrows) found in non-diabetic control models while Panel (**2**) depicts the attenuation and/or loss of the endothelial glycocalyx (arrows) by lanthanum nitrate perfusion fixation staining in the BTBR *ob/ob* diabetic model. Panel (**D**) depicts astrocyte foot process detachments from the endothelial neurovascular unit (NVU) with a nearby activated microglial cell (aMGC) and a labeled microbleed adjacent to the NVU in this low magnification image. Panel (**E**) depicts marked basement membrane thickening that is associated with the capillary NVU and note the detached astrocyte foot processes (ACfp) pseudo-colored red and the aMGC. Panel (**F**) depicts an aMGC (pseudo-colored red) that is encompassing the capillary NUV and note again the detached and retracted ACfp (drAC) (pseudo-colored cyan) with magnification ×2500 (not shown). These images display the remodeling changes that accompany the uncoupling of the NVU in brain grey matter in cortical layers III. Images in this compilation figure were modified with permission by CC 4.0 [27,32].

In contrast to the control models the *db/db* diabetic models may be characterized by an attenuation and/or loss of the tight and adherens junctions (TJ/AJ) of the endothelial cells of the NVU, which form the electron dense barrier of the blood brain barrier (BBB). Importantly, we have been able to demonstrate an attenuation and or loss of the endothelial glycocalyx in the hypoleptinemic diabetic leptin deficient BTBR *ob/ob* models [40]. We also have observed Pcfp retraction and ACfp separation and retraction from the ECs of the NVU with microbleeds. Additionally, basement membrane thickening was also noted. Interestingly, we have observed that the ramified microglial cells (rMGCs) remodel to an ameboid—activated and invasive MGC that encompasses the NVU. These remodeling changes to the NVU allow for a leaky capillary endothelial NVU with increased permeability of the BBB as well as promoting NVU uncoupling in the cortex (layer III of the grey matter and hippocampus) (Figure 6) [40,41].

The uncoupling of the NVU results in regional hypoxia and decreased cerebral blood flow (CBF) with associated impaired neurotransmission and impaired cognition. Other than the chromatin condensation in activated microglia, we do not presently know if impaired FOCM is directly involved in NVU remodeling and uncoupling; however, attenuation and/or loss of the EC TJ/AJ due to increased RONS may be due to impaired FOCM and a decrease in the antioxidant glutathione (GSH) as a result of HHcy. This increase in RONS could definitely contribute to the changes in the morphological remodeling of the NVU cells and could also increase the permeability of the NVU and its neurovascular uncoupling due to elevated levels of Hcy—HHcy (in impaired FOCM) in addition to the elevations in glucose (glucotoxicity) and oxidative—redox stress with increased RONS due to T2DM. Importantly, glucotoxicity and HHcy may act synergistically due to the increased RONS due to both glucotoxicity induced RONS and HHcy induced RONS.

### 2.2. The Endothelial Glycocalyx (ecGCx) in T2DM

The intact endothelial glycocalyx (ecGCx) is important for the vascular integrity of arteries, arterioles and capillaries. It is composed of a sugar-protein mesh, gel-like surface coating on the luminal apical polarized endothelial monolayer. This protective surface layer coating modulates the direct contact of the blood and its components (circulating leukocytes, platelets, red blood cells and larger plasma proteins) with the extracellular matrix ecGCx surface layer of the endothelial cells. The ecGCx is primarily synthesized by the endothelium with some contributions by plasma albumin, orosomucoids, fibrinogen, circulating glycoproteins and glycolipids [27,40,41,42,43,44,45,46,47,48]. Additionally, the ecGCx is anchored to the endothelial luminal plasma membranes by highly sulfated proteoglycans (syndecans and glycipans), glycoproteins (including selectins such as various cellular adhesion molecules and integrins) along with non-sulfated hyaluronan (a glycosaminoglycan) via CD44. Hyaluronan may also be free floating (unbound) or attached to the assembly proteins, such as the endothelial hyaluronan synthases as well as forming hyaluronan-hyaluronan unbound stable complexes.

**Figure 7:**
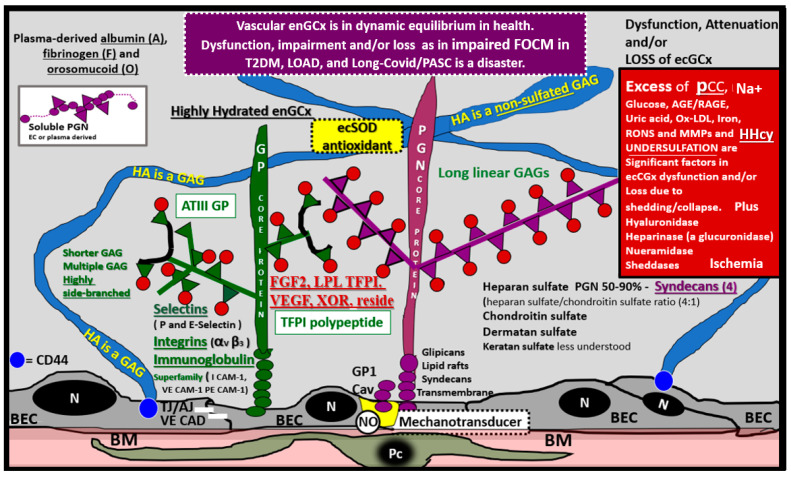
The Endothelial Glycocalyx (ecGCx). This illustration depicts the normal components of the ecGCx: a unique extracellular matrix. The ecGCx in T2DM, LOAD and Long COVID/PASC is the first component of the endothelial cell (EC) that comes into contact with the blood components of the vascular lumen. The normal components of the ecGCx include two classes of proteins that are mostly anchored proteoglycans (PGN) (purple), glycoproteins (GP) (green) and Hyaluronic acid—Hyaluronan (HA) (blue) that is an exceedingly long polymer of disaccharides that are non-sulfated glycosaminoglycans. HA may be either unattached—free floating or anchored to CD44 on the plasma membrane of ECs, or form HA–HA complexes. HA may also reversibly interact at the lumen with plasma-derived albumin, fibrinogen and soluble PGNs. The PGNs and GPs side chains consist of glycosaminoglycans (GAGs), which are covalently bound to their core proteins and are highly sulfated, which are important for giving the ecGCx its net-negative charge. The two primary PGNs are the syndecans and glypicans. The GPs consist primarily of selectins (P and E), integrins (alpha v and beta 3) and the immunoglobulin superfamily of ICAM-1, VE-CAM and PE CAM-1. Caveolae are invaginations of lipid rafts on the EC plasma membrane and contain CD44 important to anchor glycosylphosphatidylinositol (GPI) that anchor glypican-1. The GPI/glypican-1 interaction is thought to activate endothelial nitric oxide synthase (eNOS) to produce bioavailable nitric oxide (NO) via the calcium calmodulin dependent Caveolin-1 (Cav-1) protein. Note on the right-hand side of this image the numerous causes for the attenuation/shedding or loss of the ecGCx. Note that Hcy is included since it is elevated in both T2DM and Late onset Alzheimer’s disease (LOAD). Hcy may compromise the ecGCx due to its elevation, which results in hyperhomocysteinemia (HHcy), oxidative stress with elevation in reactive oxygen nitrogen species (RONS), inflammation and activation of matrix metalloproteinases. Note that T2DM is known to increase hyaluronidase. The impaired FOCM with hyperhomocyteinemia, oxidative stress and inflammation can be damaging to the ecGCx and contribute to endothelial cell activation and dysfunction with detrimental effects on the vascular tissue that predispose to increased vascular inflammation and a prothrombotic state and ischemia, which is also an inducer of ecGCx loss. Image provided by CC 4.0 [48]. *A = albumin; AGE/RAGE = advanced glycation end products and receptor to AGE; N = nucleus; ATPIII GP = antithrombin three glycoprotein; BEC = brain endothelial cell or just endothelial cell; BM = basement membrane; CAD = cadherin; CAM = cellular adhesion molecule; CD44 = cluster of differentiation 44; ecSOD = extracellular superoxide dismutase; F = fibrinogen;* *FGF2 = fibroblast Growth Factor 2; FOCM = folate-mediated one-carbon metabolism; GCx = glycocalyx; *ICAM**-1 *= intercellular adhesion molecule;* *Ox LDL = oxidized low-density lipoprotein;* *LPL = lipoprotein lipase; MMPs = matrix metalloproteinases; N = nucleus; Na^+^ = sodium; O = orosomucoids; Pc = vascular mural cell pericyte(s); PECAM-1 = platelet endothelial cell adhesion molecule-1. RONS = reactive oxygen species; TFPI = tissue factor pathway inhibitor;* *TJ/AJ = tight and adherens junctions; VCAM = Vascular cell adhesion protein; VE CAD = vascular endothelial cadherins;* *VEGF = vascular endothelial growth factor; XOR = xanthine oxioreductase*.

Importantly, mechanotransduction is accomplished by the ecGCx via the anchored proteoglycan (glypican) to the caveolae [49,50]. The ecGCx is important for both the overall barrier function of the endothelium and to the mechanotransduction of endothelial fluid shear stress. Luminal shear stress induces the production of endothelial derived nitric oxide (NO) via lipid rafts of the caveolae and the glycoproteins of the ecGCx to produce shear stress-induced nitric oxide (NO). NO production is very important for vasodilation of the aorta and arterioles via the signaling of NO to the vascular smooth muscle cells and pericytes of the capillaries. If this precious mechanism is lost due to a disturbance, attenuation, loss or shedding of the ecGCx then there will be a decrease in the quintessential bioavailable NO to signal vascular smooth muscle cells in arteries, arterioles and pericyte capillaries. An attenuation or loss of bioavailable NO promotes regional ischemia for tissue beds in all organs (Figure 7).

Recently, duPreez et al. has shared that the degree of sulfation and/or the position of sulfate groups may result in undersulfation and dysfunction of glycosaminoglycans of the proteoglycans and glycoproteins of the glycocalyx [51]. This group has also suggested that undersulfation and the improper positioning of sulfate groups may increase the susceptibility to COVID-19 and its severity, and therefore possibly LC/PASC, since we now know that the more severe cases of the acute disease predispose to increases in LC/PASC. Thus, the undersulfated glycocalyx may not only increase susceptibility to SARS-CoV-2 infections but also could result in increased inflammation, vascular permeability with shedding of the ecGCx that could additionally give rise to procoagulant and antifibrinolytic states and possible multiple organ failure as occurs in SARS-CoV-2 [51], as discussed in Section 4. Glycocalyx degradation, attenuation, shedding and or loss is gaining recognition as an important aspect in multiple diseases, including T2DM, LOAD and all forms of sepsis including COVID-19 pathophysiology and possibly even LC/PASC.

### 2.3. Aberrant Mitochondria and Impaired FOCM in T2DM

**Figure 8:**
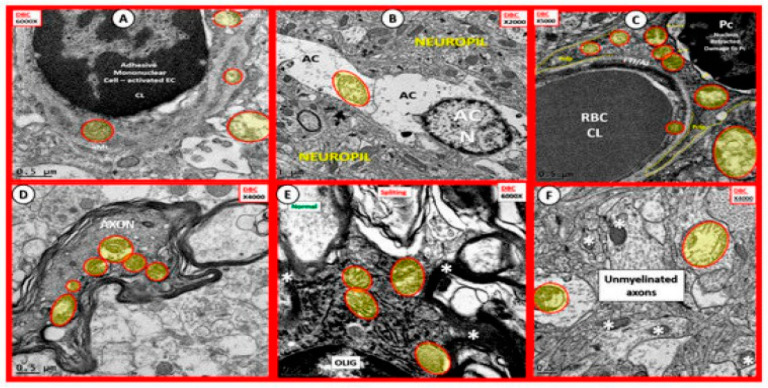
Aberrant Mitochondria in Brain Endothelial cells (EC), Astrocytes (AC), Pericytes (Pc), Myelinated and Unmyelinated Neurons and Oligodendrocytes (OLIG) in the Diabetic db/db Models. The aberrant mitochondria (aMt) are pseudo-colored yellow with red outlines in order to allow for rapid identification. Panels (**A**–**F**) demonstrate aberrant mitochondria (aMt) in each of the brain cells depicted. Panel (**A**) depicts aMt in ECs and adjacent AC foot processes. Panel (**B**) depicts an aMt within the cytoplasm of an AC. Panel (**C**) depicts multiple aMt in the cytoplasm of a Pc. Panel (**D**) depicts aMt within a myelinated neuronal axon that also demonstrates separation of its lining myelin sheath. Panel (**E**) depicts aMt in an oligodendrocyte’s cytoplasm. Panel (**F**) depicts aMt with the axoplasm of an unmyelinated neuronal axon. Note that most of the aMt are hypolucent as a result of the loss of their normal electron dense matrix proteins and they share a common feature of attenuated and or loss of their crista; they are also enlarged. Magnifications are located in the upper part of each panel and the scale bars are at the bottom. The images in this figure were modified with permission by CC 4.0 [27].

The mitochondria play an important role not only in energy production but also an important role in FOCM. In Section 1 (Figure 2) the compartmentalization of FOCM was presented showing that the mitochondrial and cytosolic FOCM operate in a parallel fashion to contribute to the formation of formate by the mitochondria and to donate SAM and tetrahydrofolate (THF) to the nucleus. Thus, the importance of the mitochondria FOCM must remain intact. If there is aberrant morphological remodeling of the mitochondria (aMt), it cannot (along with the parallel functions of the cytosol) deliver the proper molecules (formate, SAM and THF) to the nucleus in order to carry out its role in maintaining nuclear DNA and RNA modeling to allow for normal cellular function. In the female diabetic db/db models, we have observed marked abnormal remodeling of the mitochondria in each of the brain cells (Figure 8).

As noted in Figure 8, the markedly remodeled aMt in the diabetic db/db models would be incapable of proper mitochondrial FOCM function and could not function in parallel with the cytosolic FOCM in order to donate formate, SAM and THF to the nucleus to properly function. Importantly, these aMt remodeling changes could also place the nucleus at risk for epigenetic modifications and/or mutations, which could impair proper nuclear function. We previously observed this nuclear remodeling manifestation in the microglia and oligodendrocyte cells as presented in Section 2 (Figure 3) that depicted chromatin condensation within the nucleus and hyperglycemia with glucotoxicity. Additionally, T2DM is known to have elevated Hcy in humans and in the db/db diabetic models and this elevation of Hcy is thought to be a biomarker of impaired FOCM. Currently, it is not known which comes first: the abnormal structural remodeling to the mitochondria due to excess energy (hyperglycemia and elevated free fatty acid due to T2DM that remodel the Mt or if this is combined with the impaired mitochondria FOCM that could result in the Mt DNA abnormalities and loss of Mt matrix proteins as noted on the transmission electron micrographs in Figure 8; it may be that it is a bit of both working in concert to result in abnormal structure and function.

## 3. Impaired FOCM in Sporadic or LOAD

Even though the etiology of LOAD is poorly understood, it represents the most common form of dementia in the older population and may be characterized by progressive loss of memory and impaired cognition that is severe enough to interfere with activities of daily living and functioning as well as deficient measures of quality-of-life experiences. LOAD is thought to be multifactorial and polygenic and includes environmental neurotoxicant factors that result in neurodegenerative disease [4,52,53]. It may be considered as one of the most debilitating age-related neurodegenerative disorders, which affects millions of individuals worldwide. Importantly, LOAD is characterized by synaptic dysfunction, mitochondrial damage, neuroinflammation and extensive neuronal loss with associated accumulation of amyloid plaques and neurofibrillary tangles.

Homeostatic FOCM is required for the production of S-adenosylmethionine (SAM), which is the major DNA methylating agent and as previously discussed in Section 2 formate is synthesized primarily by the mitochondria. Moreover, individuals with LOAD are characterized by decreased plasma folate values in addition to increased plasma Hcy levels with an indication of impaired and/or deficient *S*-adenosylmethionine (SAM) levels in the brains of individuals with LOAD [52,53]. Furthermore, impaired FOCM has been related to LOAD in numerous studies [4,52,53,54,55,56,57,58]. Additionally, polymorphisms of genes participating in one-carbon metabolism have been associated with LOAD risk and/or with increased Hcy levels in individuals with LOAD. Studies in rodent models suggest that early life exposure to neurotoxicants or dietary restriction of folate (B9) and B12 may result in epigenetic modifications of LOAD related genes in the animal brains such as the presenilin 1 (PSEN1) gene [4,52,53]

While the brain has a range of compensatory mechanisms to maintain metabolic balance, it has been observed that these mechanisms may become overwhelmed when certain genes are abnormal, one or more of the enzymes found in FOCM is deficient and if there are deficient vitamin B9 (folate), B12 or B6 intake or uptake. This is especially true if there are co-existing elevations in Hcy levels (HHcy) that result in the reduced ability to synthetize, methylate and repair DNA, RNA and/or modulation of neurotransmission. Importantly, these aspects appear to favor the hallmarks of LOAD when combined with increased oxidative stress as in those individuals who are apolipoprotein E (ApoEε4) carriers [59,60] or those with T2DM.

### 3.1. Vascular Contributions to Cognitive Impairment and Dementia (VCID) including LOAD and Impaired FOCM

**Figure 9:**
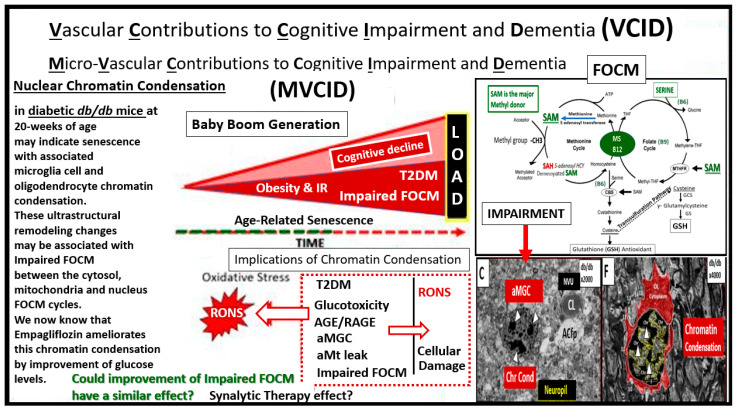
Vascular Contributions to Cognitive Impairment and Dementia (VCID), LOAD and FOCM. Our current society is aging, especially in the group referred to as the baby boom generation and age-related senescence. The aging society including the baby boomers is at a much higher risk for cerebro-cardiovascular disease and stroke especially with their comorbidities of aging such as hypertension and type 2 diabetes (T2DM). This slide focuses on T2DM, LOAD and age-related senescence that is ongoing as our population ages. This image does not portray inflammation; however, it is strongly related to an excess of RONS generated by aging, T2DM, LOAD. This figure points out the nuclear chromatin condensation of the amoeboid, aberrant and activated microglia cells (aMGC) and senescence, which highlights inflammation and its co-occurrence with oxidative stress via reactive oxygen nitrogen species (RONS). Of great concern is that non-replicative senescence can be induced by a variety of factors, including DNA damage (such as chromatin condensation), inflammation, mitochondrial dysfunction, oxidative stress (RONS) and importantly epigenetic disruption as related to impaired folate one-carbon metabolism (FOCM). The importance of impaired FOCM and each of the above listed factors may contribute to the inflammatory contribution of microglia senescence and nuclear chromatin condensation as depicted from our previous discussions in Section 2, Figure 3 and hence the reason for placing images C and F within this framework as well as illustrating the folate and methionine cycles. Since we have found numerous neurovascular unit remodeling changes in the diabetic *db/db* models, we suggest that VCID might also be considered microvascular VCID or MVCID. Impairment of the cytosolic, mitochondria and nuclear FOCM appear to be playing a role due to excessive reactive oxygen nitrogen species (RONS). Moreover, since there is obvious ongoing senescence in T2DM and LOAD it seems appropriate to ask the following question. Could improvement of impaired FOCM be a possible therapy for senescence and possibly contribute to a synalytic therapy effect? It seems appropriate to discuss this since the field of synalytic therapies is in an emerging stage of study. *AGE/RAGE = advanced glycation end products and their receptor RAGE; aMGC = aberrant-activated microglia cell; aMt = aberrant mitochondria; Chr Cond = chromatin condensation*.

Snyder et al. have introduced and discussed the role of VCID and how impaired vascular factors contribute to dementia and LOAD (Figure 9) [61].

Furthermore, Hayden et al. has recently discussed VCID [32] in relation to impaired, activated and aberrant microglia cells, but not from a standpoint of impaired FOCM so it is important to now view the role VCID in the development of LOAD and include the concept of impaired FOCM. Additionally, a paper by Snyder et al. included a distinguished group of physicians and researchers (with a total of 20 authors) who were especially keen on discussing the co-existence of vascular and LOAD molecular mechanisms and pathological findings as well as discussing the role of microinfarcts and ischemia with abnormalities of the white matter and its remodeling as we have previously noted in the 20-week-old *db/db* mice. One model developed by Sudduth et al., exists wherein they created a model for hyperhomocysteinemia by feeding wild type mice a diet deficient in vitamins B9, B12 and B6 with supplementation of excess methionine, and studied the effects of vascular dementia and LOAD [62]. Incidentally, vascular dementia is second only to LOAD as a cause of dementia and it is estimated that 40–50% of patients with LOAD may have a mixed dementia in which there are the findings of co-existing vascular dementia mixed with LOAD dementia as determined by cerebrovascular pathologic findings [63]. There are many questions to be answered in how LOAD affects small vessel function and in turn, how vascular dysfunction contributes to the molecular pathology of LOAD [64]. LOAD and vascular dementias (VAD) equal co-occurrence dementias such that LOAD + VaD + cerebral amyloid angiopathy (CAA) equals mixed dementia when one includes neuropathologic findings from autopsy [27]. Also, it has recently been shared that neurovascular disease, which includes the NVU may be one of the earliest findings in LOAD [64,65]. Moreover, the two-hit vascular hypothesis (NVU dysfunction the first hit leads to BBB dysfunction with resulting decreased cerebral blood flow and hypoxemia and is followed by hit two the neuronal remodeling that precedes dementia) has been recently placed into acceptance in addition to the other multiple LOAD hypotheses [66,67] and recently a paper with 69 authors, which discussed in detail the importance of the NVU and its constituent cells as well as other related risk factors, has been published [68].

### 3.2. Examining the Mitochondrial Cascade Hypothesis in LOAD and Impaired FOCM

There have been numerous hypotheses in regards to LOAD during the past decades including the cholinergic hypothesis, amyloid cascade hypothesis, neuroinflammation hypothesis, synaptic failure hypothesis and the two-hit vascular hypothesis. Swerdlow and Khan have recently brought forth the mitochondrial cascade hypothesis in LOAD [69]. Their hypothesis briefly proposes that the changes in mitochondria structure and function alter amyloid beta homeostasis. Importantly, this hypothesis provides strength to the discussion regarding aberrant mitochondria set forth in previous Section 2.3.

## 4. Impaired FOCM in LC/PASC

The emerging post-viral syndrome of LC/PASC in patients who have recovered from acute COVID-19 infections currently represent a growing number of individuals globally. It has been estimated that prevalence of LC/PASC ranges from 5% among non-hospitalized to 80% of those who have been hospitalized with acute COVID [70,71]. These individuals have termed themselves as being “long-haulers”, while the medical community has termed this condition LC/PASC. Currently, there is still not a standardized definition for the timeline of LC/PASC as to when it begins (somewhere between 4–12 weeks); however, these individuals do represent a failure to return to a baseline state of health after recovering from acute COVID-19. Their symptoms encompass various physical, neurologic and neuropsychiatric symptoms that may last from weeks to months following their acute infection. Typical symptoms of LC/PASC include fatigue (central/cognitive, or mental and peripheral/muscular or physical), shortness of breath, chest pain, “brain fog” (impaired cognition with difficulty in maintaining focus), sleep disorders (insomnia), fevers, gastrointestinal symptoms (diarrhea, nausea and/or vomiting), anxiety and depression, which can range from mild to incapacitating.

Recent estimates (May 2021) suggest that between 10–35% of patients that did not require hospitalization for COVID-19 might develop LC/PASC regardless of co-morbidities [72,73] and additionally of all individuals infected with SARS-CoV-2, the prevalence of LC/PASC is now thought to be from 10–30% [74]. Komaroff and Bateman are concerned that significant numbers of patients with COVID-19 might develop myalgic encephalomyelitis/chronic fatigue syndrome (ME/CFS) that is similar to LC/PASC [75]. This group calculated that roughly 10% would develop ME/CFS of approximately 25 million by the end of 2021. Importantly, if these calculations are correct, this would lead to a total of approximately 2.5 million individuals who would meet the standards for the National Academies of Sciences, Engineering and Medicine (NASEM) case definition criteria for ME/CFS in the United States, alone [75]. While the true prevalence of LC/PASC post-viral syndrome is currently unknown, it has been stated that as many as one-in-three survivors or more may develop LC/PASC [76].

While the COVID-19 pandemic has caused great morbidity and mortality across the world, many have survived its acute effects and a large number of those survivors, even those with only minor acute symptoms are now experiencing a debilitating and prolonged post-viral syndrome termed LC/PASC.

**Figure 10:**
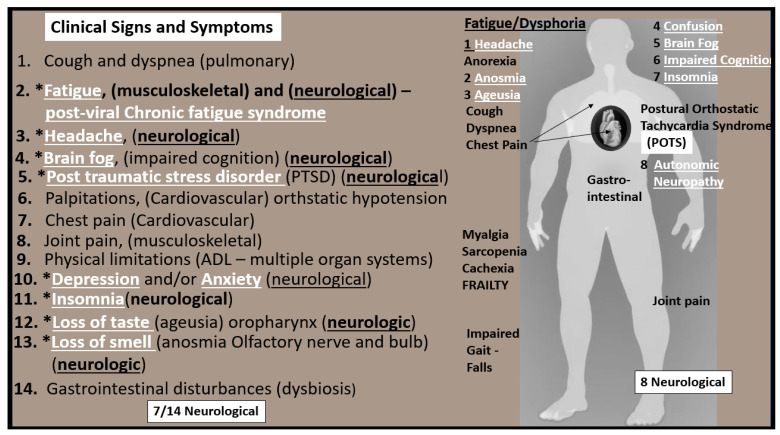
Clinical Signs and Symptoms of LC/PASC. This illustration shares at least 14 of the most common symptoms reported by LC/PASC individuals. Note that the symptoms that are related to neurological manifestations are underlined and appear in white bold font and preceded by asterisks. Additionally, note that fatigue and dysphoria are at the top of the list at the heading of the ‘standing man’ to the right-hand side of this figure as one of most aggravating clinical signs and symptoms of the remaining neurological manifestations. Further, note that this ‘standing man’ is portrayed as being a muscular robust individual that is not frail or old. In many cases, the individuals who have LC/PASC are very healthy and even young.

Post-COVID LC/PASC multispecialty clinics have emerged that are associated with large hospital systems in larger cities, which are able to provide multiple specialists that will be required to address each possible dysfunction in multiple different organ systems since SARS-CoV-2 affects nearly all systems in the body, including the brain. Importantly, many of these patients will require a program of progressive physical therapy to help regain their pre-COVID physical activity and endurance. In contrast, the burden of LC/PASC patients’ health care in the more rural regions, will be largely managed by the patient’s family physicians and their supportive nurse practitioners and primary care providers in outlying clinics who will not have the benefit of a multi-specialists care team of providers [73]. Patients with LC/PASC suffer from a multitude of symptoms as previously discussed; however, approximately 50% are directly or indirectly related to the brain or central nervous system (CNS); therefore, we have chosen to focus on the CNS to discuss the implications of impaired FOCM in LC/PASC. For example, of the 14 most common symptoms that are associated with LC/PASC, seven appear to be related to the brain and consist of fatigue, headache, brain fog, post-traumatic stress disorder, depression and/or anxiety, insomnia and loss of taste or smell (Figure 10) [48].

This problem with LC/PASC is very real and may continue to grow without any current treatment that seems to fit all individuals with LC/PASC.

### Compromised FOCM in LC/PASC: Importance of HHCY and Deficient Micronutrients (Vitamins B12 and B9)

Individuals with COVID-19 frequently have not had the best nutrient intake, whether they were treated in a hospital (in ICU or non-ICU beds) or at home. Thus, these individuals’ micronutrient intake of B vitamins may be diminished in LC/PACS. Additionally, SARS-CoV-2 is certainly capable of creating an assault on one-carbon metabolism [77]. As SARS-CoV-2 undergoes rapid replication during the viremia stage, it will hijack the hosts cells normal mechanism for supplying methyl groups [78]. This will significantly stress the host cells FOCM and may result in not only increased demand but also will impair the supply of methyl-groups for the host cell in addition to the underlying increase in oxidative stress and RONS accumulation due to impaired FOCM and HHcy [77]. Additionally, during this hijacking mechanism, SARS-CoV-2 will result in the depletion of serine and glutathione (GSH) with an increase in Hcy. LC/PASC has numerous overlapping symptoms with pernicious anemia (PA) and ME/CFS, which are both B12-responsive syndromes [79]. For these reasons, one can now appreciate why those with LC/PASC may be at risk for having depleted or impaired FOCM as we begin to better understand FOCM [48]. Additionally, the chronic inflammation associated with the peripheral cytokine/chemokine and CNS cytokine/chemokine excess due to SARS-CoV-2 will place these LC/PASC individuals at an increased risk of oxidative stress with an increase of RONS) [48]. Importantly, this oxidative stress will increase the risk of oxidation to the redox sensitive co-factor vitamin B12 (cobalamin I) to be oxidized to cobalamin II, which will not run the MS reaction. This will result in the impaired ability for methionine synthase (MS) to convert Hcy to methionine resulting in HHcy in the methionine re-methylation cycle and the conversion of methyl-THF to THF in the folate cycle (Figure 1). [48,77,80,81].

**Figure 11:**
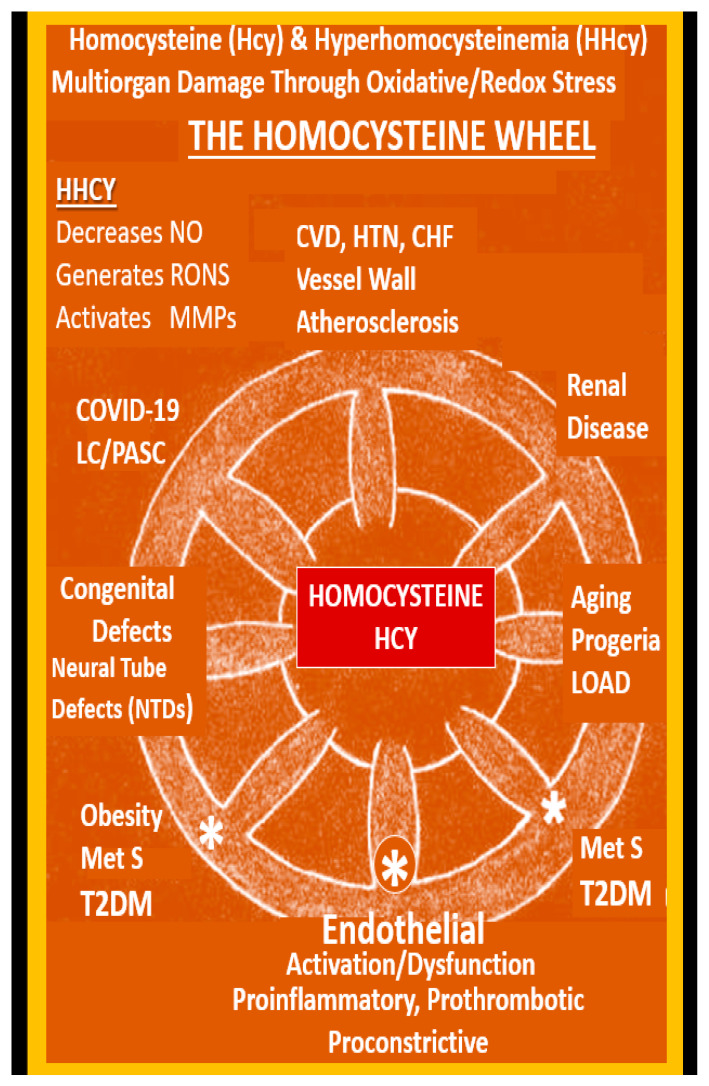
The Homocysteine Wheel. This illustration depicts the central importance of homocysteine (Hcy) in its hyperhomocysteine (HHcy) state as it relates to the multiorgan tissue damage and associated multiple clinical diseases, which now includes the COVID-19 post-viral syndrome of LC/PASC. HHcy generates damage to proteins, lipids and nucleic acids as result of oxidative redox stress and the generation of excessive reactive oxygen nitrogen species (RONS). In HHcy, Hcy undergoes autoxidation, formation of Hcy mixed disulfides, interaction of Hcy thiolactones and protein homocysteinylation reactions, which result in damage and dysfunction to proteins, tissues and organs. Note the large asterisk at the central bottom of this illustration where the effects of HHcy promotes the uncoupling of the endothelial nitric oxide synthase enzyme (eNOS) by oxidizing the tetrahydrobiopterin (BH4) co-enzyme of eNOS and thus promotes an even further production of superoxide while it concurrently decreases the bioavailability of nitric oxide (NO), thus important to normal endothelial cell dysfunction. As a result, the endothelium undergoes activation and dysfunction and becomes a proinflammatory, prothrombotic endothelium due to increased generation of RONS. While this review has focused primarily on COVID-19 and LC/PASC, this illustration demonstrates that HHcy affects many other tissues, organs and clinical diseases. This modified image is reproduced with permission by CC 4.0 [6]. *CHF = congestive heart failure; COVID-19 = coronavirus disease-19; CVD = cebro-cardiovascular disease; HTN = hypertension; MetS = metabolic syndrome; MMPs = matrix metalloproteinases; NTD = neural tube defects; LC/PASC = Long COVID/*post-acute sequelae of SARS-CoV-2; *LOAD = late-onset Alzheimer’s disease; T2DM = type 2 diabetes mellitus*.

**Figure 12:**
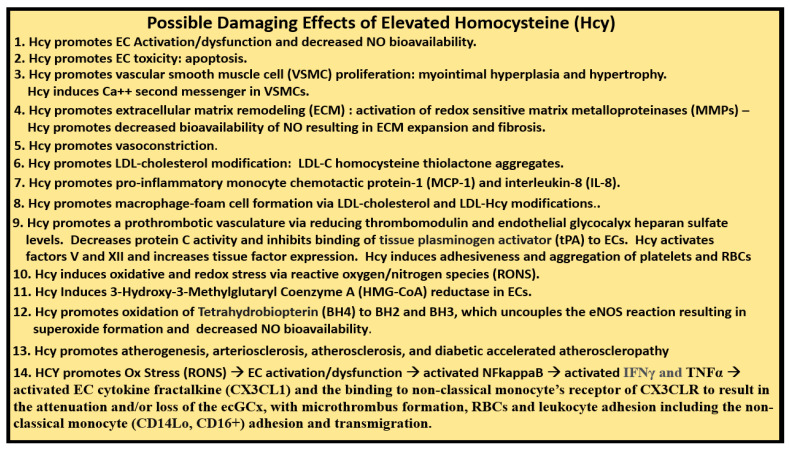
Possible Damaging Effects of Elevated Homocysteine (Hcy). There are at least 14 possible damaging effects of hyperhomocysteinemia (HHcy) to cells, tissues and organs. The possible damaging effects (1–13) are supported in reference [6], while the possible damaging effect of HHcy in number 14 is developed in the current review. Additionally, circulating monocytic SARS-CoV-2 spike protein remnants may also induce toxic cytokine/chemokine production in endothelial cells. *CD14lo = cluster of differentiation 14 low; CD16+ = cluster of differentiation 16 positive; EC = endothelial cell; ecGCx = endothelial cell glycocalyx; eNOS = endothelial nitric oxide synthase; IFNγ = interferon gamma; LDL = low-density lipoprotein cholesterol; NO = nitric oxide; Ox = oxidative stress; RONS = reactive oxygen nitrogen species; NFkappaB = nuclear factor kappa B; RBCs = red blood cells; TNFα = tumor necrosis factor alpha*.

Impaired FOCM in LC/PASC may be associated with an elevation of the biomarker Hcy with resulting HHcy and its possible damaging effects on tissues and organs (Figure 11 and Figure 12).

Hcy has not been evaluated in many COVID-19 studies to date; however, it has recently been proposed as a potential predictive biomarker for COVID-19 infection severity [11,82]. Importantly, Ponti and colleagues have recently demonstrated in a study of 313 COVID-19 patients that Hcy was a predictive marker for hospitalized COVID-19 patients’ outcomes [83]. They were able to demonstrate that plasma Hcy levels correlated significantly both as a continuous and dichotomic value with an optimal cut-off of 16 μmol/L. Additionally, they pointed to a previous study, which included 273 COVID-19 patients wherein HHcy was reported to be predictive for computed tomography-imaging lung damage progression. Furthermore, this group pointed out that HHcy was attributed to many viral infections, including human hepatitis, papilloma and immunodeficiency viruses. Additionally, the C677T allele of the methylenetetrahydrofolate reductase (MTHFR) gene appears to be correlated with COVID-19 [84].

Unfortunately, at the time of this manuscript preparation authors were unable to find any publications regarding the role of HHcy in patients with LC/PASC; however, it is felt that in due time these data may be forthcoming since there is such a strong overlap of LC/PASC with ME/CFS that is known to be associated with elevated levels of Hcy [48,77,78,79,80].

## 5. Future Possible Treatment Options for LC/PASC

Just as there are no current precise definitions of the exact time frames to consider those who are suffering from LC/PASC, there are also no known treatment protocols that have been successfully studied in placebo verses treatment-controlled trial studies in LC/PASC. However, in the United States, the United States Congress has provided 1.15 billion dollars in funding to be made available over four years for the NIH to support research into the prolonged health consequences of SARS-CoV-2 infection [85]. As a result of this novel post viral syndrome due to SARS-CoV-2, it may be some time before we will know the exact biomechanisms that are responsible for LC/PASC as well as any specific treatment paradigm. Therefore, any treatment modality that may be discussed at this point in time may be deemed speculative.

However, Patterson et al. seem to be leading the way for those suffering from LC/PASC with the use of artificial intelligence, indentified algorithms and precision medicine in identifying a LC/PASC—‘long-haulers index’ that appears to be both reliable and reproducible [85,86]. This group has shown that SARS-CoV-2 leaves behind spike (S1) protein material in the organs affected by COVID-19. Subsequently, they have identified a non-classical antigen presenting monocyte (CD14Lo, CD16+) that is responsible for the uptake of these (S1) spike remanent proteins resulting in the activation of these non-classical monocytes [86,87]. They also found that these non-classical monocytes were significantly elevated in PASC patients up to 15 months post-acute infection compared to healthy controls. Further, they have shown that these patrolling, roaming proinflammatory and prothrombotic non-classical monocytes circulating cells are capable of adhering to endothelial cells via the binding of these non-classical monocytes to the ECs fractalkine—CX3CL1 [87]. This would cause vascular inflammation (endotheliitis–vasculitis) throughout the body as a result of the activated/dysfunctional ECs due to the adhesion of non-classical monocytes, which promote a proinflammatory, pro-oxidative and prothrombotic endothelium [86,87]. Their findings support the use of CCLR5 antagonists in order to prevent the migration and adherence of these non-classical monocytes to the endothelium due to the elevated levels of CCL5 (chemokine (C-C motif) ligand 5)—RANTES (regulated on activation, normal T cell expressed and secreted) in PASC. Additionally, these authors have suggested that interruption of the CX3CR1/EC fractalkine pathway would be a potential therapeutic target to reduce the survival of S1-containing non-classical monocytes and the associated vascular inflammation [86,87]. In summary Patterson’s group have suggested that therapeutics involving the blockade of 1) inflammation (low-dose prednisone), 2) CCL5/RANTES activation (CCR5 antagonists) and 3) endothelial cell fractalkine cycle activation (statins) may be successful as identified in their long-haulers index (IFN-γ + IL-2/CCL4 − MIP-1β) they have recently described and discussed in detail [86,87].

Regland et al. has previously (2015) demonstrated a positive response to vitamin B12 by injection and oral folic acid in myalgic encephalomyelitis and fibromyalgia individuals, which has been clinically likened to LC/PASC [79]. Moreover, Hayden has shared his own personal experiences in the treatment of a post-viral fatigue syndrome in herpes simplex virus type 1 (HSV-1) encephalitis and in a small community outbreak of human cytomegalic virus (hCMV) in addition to Epstein–Barr virus (EBV) infections [48]. He has previously utilized anti-inflammatory methylprednisolone and B12 by injection and oral B9 supplementation treatment to improve impaired FOCM [48]. Interestingly, this treatment protocol to reduce inflammation and improve impaired FOCM could be synergistic to Patterson’s group discussed in the previous paragraph [86,87].

**Figure 13:**
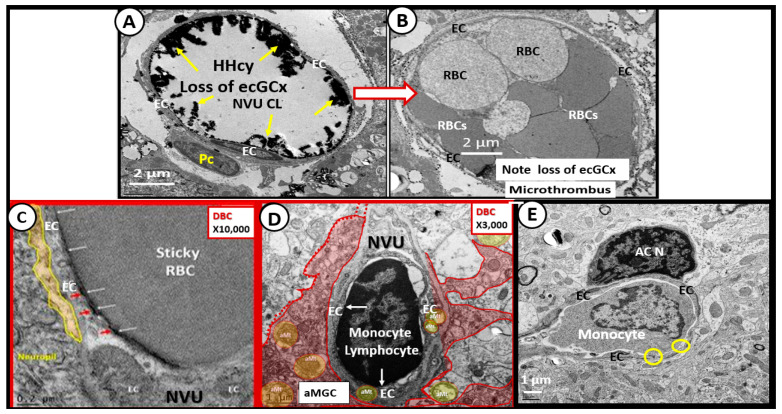
Impaired FOCM and Elevated Hcy (HHcy) Results in an Activated Endothelium that Promotes Activated Fractalkine (CX3CL1). This collection of transmission electron micrographs from the female diabetic *db/db* models and diabetic BTBR *ob/ob* models in cortical grey matter (layer III) allow for illustrative and representative images that may also be present in Long COVID/post-acute sequelae of SARS-CoV-2 (LC/PASC). Impaired FOCM is associated with hyperhomocysteinemia (HHcy) and results in an activated endothelium with an attenuation and/or loss of endothelial glycocalyx (ecGCx) (yellows arrows pointing to lanthanum nitrate positively stained ecGCx) in the control model (panel (**A**)), which predisposes to the attenuation and/or loss of the ecGCx in diabetic BTBR *ob/ob* models and the development of microclots and microthrombi (panel (**B**)). Additionally, impaired FOCM with HHcy is associated with endothelial activation and dysfunction resulting in adhesion of red blood cell(s) (RBCs) (panel (**C**)) and adhesion of leukocytes such as lymphocytes (panel (**D**)) and monocytes (which may include the non-classical monocyte (CD14Lo, CD16+) (panel (**E**)). Magnifications vary and scale bars are included. This modified multi-panel figure is reproduced with permission by CC 4.0 [6,27,32,41].

Thus, the question arises as to how improving impaired FOCM might be examined from a mechanistic approach in treating LC/PASC. Elevations in Hcy (HHcy) are thought to be an important biomarker of impaired FOCM and it is known that HHcy is detrimental to the proper function of ECs and to the normal functions of multiple tissues and organ systems (Figure 11 and Figure 12). HHcy is known to play a detrimental role via its capability to induce oxidative stress (increase in RONS) in ECs that result in EC activation and dysfunction and activation of nuclear factor kappa-light-chain-enhancer of activated B cells (NFkappaB—NF-κB) [88]. Additionally, it is known that HHcy via its increase in oxidative stress (RONS) and its activation of NFkappaB may not only activate ECs but HHcy is also known to increased fractalkine (CX3CL1—chemokine) expression [89]. This could also contribute to the attraction of non-classical monocytes discussed in the previous paragraphs by Patterson’s group and allow for the simplified progressive sequence of events as follows: Impaired FOCM →, HHcy →, Ox Stress (RONS) →, EC activation/dysfunction →, activated NFkappaB →, activated interferon gamma (IFNγ) and tumor necrosis alpha (TNFα) →, activated EC cytokine fractalkine (CX3CL1) and the binding of the non-classical monocyte’s (CD14Lo, CD16+) receptor of CX3CLR to result in the attenuation and/or loss of the ecGCx, with microthrombus formation, red blood cell and leukocyte adhesion, including the non-classical monocyte (CD14Lo, CD16+) adhesion (Figure 13) [86,87,88,89].

Additionally, it is interesting to consider the epigenetic changes within the nuclei that may be involved as a result of impaired FOCM that were discussed and presented previously in Section 2 (Figure 3) regarding nuclear chromatin condensation in activated microglial cells and oligodendrocytes. Interestingly, Corley et al. have revealed an epigenetic signature associated with severe COVID-19 and there may be other epigenetic changes found to correlate with LC/PASC in the future [90].

Thus, the early recommendations for the possible treatments of LC/PASC being placed forward in this section may be synergistic. We must be open to other future possible findings and recommendations as they emerge in this field of LC/PASC research.

## 6. Conclusions

This review has demonstrated the important role of FOCM in health and in specific disease states via the discussion of impaired FOCM in T2DM, LOAD and the emerging post-viral syndrome of LC/PASC. The important role of FOCM in homeostasis and health depends on an adequate nutritional uptake and absorption and/or supplementation of the essential water-soluble vitamins B6, B9 and B12. B vitamin (B6, B9 and B12) intake and absorption play a crucial role in FOCM, which provides homeostasis and health. The cellular compartmentalization of FOCM (cytosolic, mitochondria and nuclear regions) that function in parallel and in unison is a complex set of cycles. These cycles are interconnected and interdependent biochemical pathways driven by folate and methionine cycles with their essential vitamin B co-factors to generate and donate methyl groups as presented in this review. These methyl groups are utilized for their use in nuclear deoxyribonucleic acid DNA and ribonucleic acid RNA synthesis, amino acid homeostasis, antioxidant generation and epigenetic regulation in addition to the production of energy sources via the mitochondria discussed in Section 1 (Figure 1 and Figure 2).

From a clinical perspective one should view HHcy as a biomarker and a risk for impaired FOCM. Clinicians and researchers should therefore view HHcy as a definite ‘red flag’ for the existence of underlying impaired FOCM or a genetic abnormality. Indeed, impaired FOCM is often viewed through the lens of HHcy since elevated levels of Hcy are often one of the most common abnormalities that is present in clinical diseases associated with impaired FOCM. The bottom line is that measuring Hcy is a useful test for the clinician, as it carries important prognostic information in regards to impaired FOCM in disease.

Correcting impaired FOCM will help us to better understand the therapeutic modulation of T2DM, LOAD and LC/PASC. There still remains a great deal of research to be accomplished in this field of impaired FOCM and the future treatment of LC/PASC. It is hoped that this review will help to better understand impaired FOCM in clinical diseases and will aid in understanding some of the gaps in knowledge.

## Data Availability

Data and materials will be provided upon reasonable request.
